# Activated protein C increases sensitivity to vasoconstriction in rabbit *Escherichia coli *endotoxin-induced shock

**DOI:** 10.1186/cc4858

**Published:** 2006-03-15

**Authors:** Eric Wiel, Marion Elizabeth Costecalde, Gilles Lebuffe, Delphine Corseaux, Brigitte Jude, Régis Bordet, Benoît Tavernier, Benoît Vallet

**Affiliations:** 1 EA 1046, Laboratory of Pharmacology, University Hospital of Lille, France; 2 Federation of Research in Anesthesiology and Intensive Care Medicine, University Hospital of Lille, France; 3 Prehospital Emergency Department (SAMU 59), University Hospital of Lille, France; 4 EA 2693-INSERM-ESPRI, Laboratory of Hematology, University Hospital of Lille, France

## Abstract

**Introduction:**

The aim of this study was to investigate the effects of activated protein C (aPC) on vascular function, endothelial injury, and haemostasis in a rabbit endotoxin-induced shock model.

**Method:**

This study included 22 male New Zealand rabbits weighing 2.5 to 3 kg each. *In vitro *vascular reactivity, endothelium CD31-PECAM1 immunohistochemistry, plasma coagulation factors and monocyte tissue factor (TF) expression were performed 5 days (D5) after onset of endotoxic shock (initiated by 0.5 mg/kg intravenous bolus of *Escherichia coli *lipopolysaccharide (LPS)) with or without treatment with aPC injected as an intravenous 2 mg/kg bolus 1 hour after LPS (LPS+aPC group and LPS group, respectively).

**Results:**

LPS decreased the sensitivity to phenylephrine (PE) in aortic rings without endothelium (E-) when compared to E- rings from the control group (*p *< 0.05). This was abolished by N^G^-nitro-L-arginine methyl ester and not observed in E- rings from aPC-treated rabbits. Although aPC failed to decrease monocyte TF expression in endotoxinic animals at D5, aPC treatment restored the endothelium-dependent sensitivity in response to PE (2.0 ± 0.2 μM in rings with endothelium (E+) versus 1.0 ± 0.2 μM in E- rings (*p *< 0.05) in the LPS+aPC group versus 2.4 ± 0.3 μM in E+ rings versus 2.2 ± 0.2 μM in E- rings (*p* value not significant), in the LPS group). Endotoxin-induced de-endothelialisation was reduced by aPC at D5 (28.5 ± 2.3% in the LPS+aPC group versus 40.4 ± 2.4% in the LPS group, *p *< 0.05).

**Conclusion:**

These data indicate that aPC increased the sensitivity to a vasoconstrictor agent (PE) associated with restoration of endothelial modulation, and protected against endothelial histological injury in endotoxin-induced shock. It failed to inhibit TF expression at D5 after LPS injection.

## Introduction

Septic shock is often associated with vascular damage, haemostasis activation and development of disseminated intravascular coagulation leading to multiple organ dysfunction and death [1]. In such conditions, morphological and functional endothelial abnormalities are considered to be involved in the development of circulatory failure [2-4].

Morphological injuries are characterized by endothelial detachment and denudation reaching approximately 20% to 35% of the endothelial surface [5-7]. They are associated with coagulation activation through monocyte tissue factor (TF) expression [1,7], and with impaired contractile induction of endothelial modulation [7]. Furthermore, sepsis alters the nitric oxide (NO) pathway, with a reduction of endothelial constitutive NO synthase (NOS) expression and overexpression of vascular smooth muscle cell inducible NOS (iNOS). Overall, these phenomena contribute to the refractory hypotension and altered tissue perfusion observed during septic shock. In human volunteers, it was demonstrated that endotoxin injection is associated with prolonged coagulation activation and endothelial injury [8]. In the rabbit endotoxin shock model, we reported that endothelial injuries and monocyte TF expression are sustained, persisting longer than five days after a single injection of lipopolysaccharide (LPS) [7-12]. Persistence of inflammatory activation via the nuclear factor (NF)-κB pathway could explain, at least in part, the prolonged endothelial and monocyte alterations. The anatomical and functional injuries were observed to be corrected approximately 21 days after LPS injection [7].

This diffuse vascular injury associated with the triggered blood coagulation cascade results in microvascular thrombosis and disseminated intravascular coagulation responsible for multiple organ failure [13]. The anticoagulant protein C (PC) pathway controls microvascular thrombosis, limiting the coagulation response to injury [14]. Once bound to thrombomodulin, thrombin loses its procoagulant properties by its inability to act upon fibrinogen as a substrate for conversion to fibrin, and turns into an anticoagulant by activating PC. Activated PC (aPC) inactivates the coagulation cofactors Va and VIIIa through proteolytic degradation, thereby limiting thrombin generation. aPC produces then anti-thrombotic, pro-fibrinolytic and anti-inflammatory activities through several different mechanisms [15].

During severe sepsis, PC is consumed by the process of coagulation triggered by endothelial and/or monocyte TF expression, and its plasma level is lowered. This correlates with a higher mortality rate [16]. Moreover, endothelial microvascular injury is associated with functional alteration of endothelial thrombomodulin and a loss of PC activation. This is the rationale for the use of aPC, and not only PC, as a therapeutic agent for severe sepsis.

Therefore, both anti-inflammatory and anti-thrombotic actions are of interest when studying how aPC helps prevent endothelium damage and monocyte TF expression in septic shock. This study was conducted to investigate the long-term influence of aPC on endothelial function in a well-characterized rabbit endotoxin-induced shock model [7-12].

## Materials and methods

### Study protocol

The animal experiments were approved by the French Agricultural Office for the care of animal subjects, and the care and handling of the animals were in agreement with the European legislation for animal research.

We used 22 male New Zealand White rabbits, weighing 2.5 to 3 kg each, obtained from the Charles River Laboratory (St Aubin-lés-Elbeuf, France). Animals were maintained throughout on a standard rabbit chow diet with 100 g of food per day and water *ad libitum*.

For the endotoxin animals, conscious animals were rapidly injected intravenously via a marginal ear vein with 0.5 mg/kg body weight of purified LPS endotoxin (*Escherichia coli *serotype O55:B5 from a single batch; Sigma Chemical, St Louis, MO, USA).

Animals were randomly assigned to one of the four following groups: rabbits in the control (CTRL) group (*n *= 6) received normal saline; those in the LPS group (*n* = 6) received LPS alone; those in the LPS+aPC group (*n *= 6) received aPC (2 mg/kg) as a single bolus injection 1 hour after the LPS injection; and 4 rabbits received aPC alone 1 hour after saline injection in the same condition. All animals were sacrificed at 5 days (D5) after LPS or saline injection under general anaesthesia. The number of rabbits per group was chosen on the basis of our previous studies demonstrating that at least four to six animals per group were necessary to show statistical differences in the analyzed parameters [7-12].

We used recombinant human aPC because a previous study demonstrated that its action on endothelial protein C receptor (EPCR) was the same regardless of the animal species used, with the half-life differing from one species to another [17]. A dose of 2 mg/kg aPC was administered 1 hour after LPS because Jackson and colleagues showed that aPC given to dogs at a dose of 1 mg/kg/h for 2 hours was efficacious [18]. We injected aPC as a single intravenous bolus since administration by infusion would have needed the placement of a catheter in the anesthetized rabbits, and this procedure would have caused modification of the haemodynamic condition.

Arterial blood gas analysis was performed four hours after LPS or saline injection. At D5, hematological and coagulation parameters were measured in all groups. The body weight was assessed at D5 for each animal. *In vitro *vascular reactivity and endothelium CD31-PECAM1 immunoreactivity were obtained at D5.

### *In vitro *vascular reactivity

The descending abdominal aorta was removed rapidly by laparotomy under general anesthesia (pentobarbital, 30 mg/kg; Specia, Paris, France) and immersed in iced oxygenated Krebs-Henseleit solution of the following composition: 118 mmol/l NaCl, 4.6 mmol/l KCl, 27.2 mmol/l NaHCO_3_, 1.2 mmol/l MgSO_4_, 1.2 mmol/l KH_2_PO_4_, 1.75 mmol/l CaCl_2_, 0.03 mmol/l Na_2 _EDTA and 11.1 mmol/l D-glucose (pH 7.35 to 7.45). Intravenous heparin (500 IU/kg; Panpharma, Fougéres, France) was given before removal of the aorta to prevent coagulation. Vessels were cleaned of surrounding fat and connective tissue and cut into rings 3 to 4 mm long. Four rings were sectioned from each aorta. Two rings of each aorta were functionally denuded of endothelium by lightly rubbing the luminal wall with a wooden applicator. As previously described [19], all rings were mounted progressively under 8 g of resting tension (previously determined as the optimal point of their length-tension relationship) on stainless hooks in organ chambers (Radnoti Glass Technology, Monrovia, CA, USA) filled with 40 ml warmed (37°C) and oxygenated (95% oxygen/5% CO_2_) Krebs-Henseleit solution. Rings were connected to force transducers, and changes in isometric force were recorded continuously. The output from the transducers was amplified by signal conditioners and sent to an Intel 486-based computer for analog-to-digital conversion. After an equilibration period of 1 hour, the presence or absence of functional endothelium was verified by addition of acetylcholine (ACh; 3.10^-5 ^mmol/l; Sigma Chemical) to rings precontracted with phenylephrine (PE; 3.10^-7 ^mmol/l; Sigma Chemical). After a new 30 minute stabilization period at the resting tension, cumulative concentration-response curves were determined for PE (10^-9 ^to 3.10^-5 ^mmol/l). The presence of a vascular smooth muscle cell iNOS was pharmacologically determined by performing the same protocol in the presence of N^G^-nitro-L-arginine methyl ester (L-NAME; 3.10^-6 ^mmol/l; Sigma Chemical) in vessels without endothelium. Endothelium-derived vascular reactivity was assessed by application of the following: the receptor-dependent endothelium-dependent vasodilator agonist ACh (10^-9 ^to 3.10^-5 ^mmol/l); the receptor-independent endothelium-dependent vasodilator agonist calcium ionophore A23187 (10^-9 ^to 3.10^-6 ^mmol/l; Sigma Chemical); and the endothelium-independent vasodilator sodium nitroprusside (10^-9 ^to 3.10^-5 ^mmol/l; Sigma Chemical). PE, ACh, sodium nitroprusside and L-NAME were dissolved in deionized water.

### Immunohistochemical staining of vascular endothelium

Aortic segments were fixed with paraformaldehyde 4% and then cryoprotected by immersion in sucrose 30%. Tissues were embedded in optimal cutting temperature, frozen in isopentane and stored at -80°C. Tissue sections were cut 6 μm thick. The endothelial cell layer was stained by using an antibody against the endothelium-specific intercellular adhesion molecule CD31-PECAM1. Briefly, frozen sections were air-dried for 1 hour, incubated with peroxidase blocking reagent, rinsed in PBS for 10 minutes, and blocked with 10% horse serum in PBS for 10 minutes. The sections were then incubated at 37°C overnight with a mouse-prepared monoclonal primary antibody to CD31 (Dako, Carpinteria, CA, USA) diluted 1:20 in PBS. After three washings in PBS, an antimouse biotinylated secondary antibody was applied for 1 hour. The sections were washed with PBS and then incubated with avidin-biotin-peroxidase preformed complex (Vectastain Elite ABC Peroxydase kit, Vector Laboratories, Burlingame, CA, USA) for 1 hour. The peroxidase activity was revealed by using hydrogen peroxide and diaminobenzidine as a chromogen. Finally, sections were counterstained with hematoxylin and mounted with Permount (Fisher Scientific, Elancourt, France). In each experiment, negative controls without the primary antibody were included to check for nonspecific staining.

For quantification of endothelial injury, three non-consecutive cross sections per aortic segment were photomicrographed microscopically (Axioskop 20; Zeiss, Le Pecq, France). After photographic reconstruction of each tissue section, each picture was digitalized for computerized analysis (Color Image 1.32 Software). The surface area of endothelial cell injury (including the three types subendothelial vacuolization, detachment of endothelial cells and endothelial denudation) was measured and expressed as percentage of total circumference of each section.

### Hematological and coagulation studies

#### Hematological and coagulation variables

At D5, blood was sampled under sterile conditions from the ear artery. Samples collected on EDTA were used for blood cell counts (Coulter MAXM; Beckman Instruments, Fullerton, CA, USA). The total white blood cell counts were verified manually. Peripheral blood smears for differential white cell counts were stained with May Grünwald Giemsa. Each count was performed by three investigators, who were blinded to the treatment allocation. Factor II, V and VII+X levels were determined by an automated clotting assay (STA; Stago, Asnières, France) by using calcified rabbit brain thromboplastin and human factor deficient plasma (Stago). Prothrombin index was measured by an automated clotting assay by using calcified rabbit brain thromboplastin (Stago). Fibrinogen levels were measured by the Clauss technique (Biomérieux, Lyon, France).

#### Isolation of mononuclear cells, cell culture and TF activity assay

The mononuclear cells were isolated by gradient centrifugation (MSL, density = 1.077 ± 0.001; Laboratories Eurobio, Les Ulis, France), washed two times, and resuspended in RPMI 1640 (3 × 10^6 ^cells/ml; GIBCO Life Technologies, Eragny, France). Cell viability was >98% as assessed by the trypan blue test. All reagents, test tubes and culture supplies used were free of endotoxin, as determined by the chromogenic limulus amebocyte lysate assay. The sensitivity of this assay was 0.025 endotoxin units/ml. Aliquots of cell preparations (3 × 10^6 ^cells/ml) suspended in RPMI 1640 without fetal calf serum were cultured for 16 hours at 37°C in a humidified 5% CO_2 _atmosphere, with or without stimulation by endotoxin at 1 μg/ml, which corresponded to 5,000 endotoxin units/ml (*E. coli *055:B5, Sigma Chemical); these are referred to as stimulated and unstimulated cells, respectively. By the end of the incubation period, mononuclear cells were resuspended and frozen at -80°C.

TF activity was determined with a modified amidolytic assay [20,21]. Briefly, lysed cell suspensions (50 μl) were incubated at 37°C in a microtiter plate (2 minutes) and mixed with 0.25 mol/l CaCl_2 _(50 μl) (3 minutes of incubation) and prothrombin concentrate complex (Laboratoire de Fractionnement et des Biotechnologies, Les Ulis, France) as a source of factor VII (50 μl, 3 UI/ml) and factor X (6 UI/ml). After addition of 50 μl of the chromogenic substrate S2765 (Biogenic, Maurin, France), the change in optical density at 410 nm was quantified with a microplate reader and converted to units of TF activity from log-log plots of serial dilutions of rabbit brain thromboplastin (Néoplastine CI Plus; Diagnostica Stago, Asnières, France). Arbitrarily, 1 ml of thromboplastin was assigned a value of 1,000 U/ml of TF. Results were expressed as mU/1.5 × 10^5 ^mononuclear cells.

### Statistical analysis

Results are presented as mean ± standard error of the mean. Hematological and coagulation data were compared using the unpaired Student's *t *test. The concentrations of agonist causing half-maximal contraction or relaxation (EC_50_) were calculated by using nonlinear semilogistic regression analysis. EC_50 _were compared using the Mann-Whitney test. Relaxation to the vasodilator agents is expressed as percentage reduction of the maximal contraction to PE. Mean intergroup differences were tested by repeated measures analysis of variance (ANOVA), followed by Scheffé's least-significant-difference test. Significance was accepted at *p *< 0.05.

## Results

### *In vivo *parameters

Because all animals were killed at D5, five-day survivors before sacrifice were considered permanent survivors. No death was observed in CTRL and aPC groups. The mortality rate was similar both in the LPS group and the LPS+aPC group (16.7%; 1 death/6), with rabbits dying within the first 4 hours following LPS injection. Compared with the baseline values, there was a significant body weight loss at D5 in LPS-treated animals of 13.9 ± 2.1% in the LPS group and 11.1 ± 2.9% in the LPS+aPC group (not significant versus the LPS group).

### *Ex vivo *measurements

#### Arterial blood-gas analyses

Metabolic acidosis confirmed endotoxic shock at H4 (pH = 7.3 ± 0.1, bicarbonate = 9.6 ± 1.5 mmol/l, PaCO_2 _= 17.6 ± 1.2 in the LPS group versus pH = 7.4 ± 0.0, bicarbonate = 25.5 ± 1.1 mmol/l, PaCO_2 _= 40.9 ± 1.5 in the CTRL group; *p *< 0.05 for all parameters). In the aPC group, the results were pH = 7.5 ± 0.0, bicarbonate = 25.7 ± 0.7 mmol/l, PaCO_2 _= 32.5 ± 1.6; *p *< 0.05 for pH and PaCO_2 _versus CTRL group. No difference was observed between the LPS+aPC and LPS groups (LPS+aPC group, pH = 7.3 ± 0.1, bicarbonate = 8.0 ± 2.0 mmol/l, PaCO_2 _= 14.5 ± 3.5; not significant versus the LPS group).

#### Hematological and coagulation parameters

Effects of *in vivo *LPS administration on hematological and coagulation parameters at D5 are presented in Tables 1 and 2, respectively. aPC alone was responsible for a trend towards a decrease in leukocytes when compared to the CTRL group (Table 1). For coagulation variables, no difference was observed between groups for the value of the prothrombin index (Table 2). LPS increased fibrinogen and the plasma concentrations of factor II and factor VII+X when compared to the CTRL group (Table 2). These alterations were not prevented by aPC administration (Table 2).

#### Monocyte TF expression at D5

**Figure 1:**
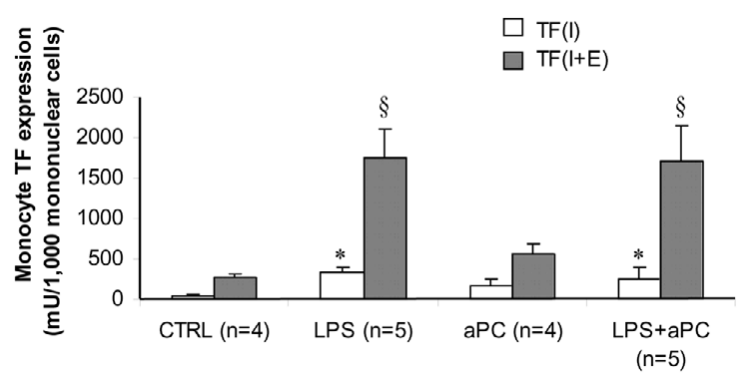
**Expression of monocyte tissue factor** (TF) at day 5 with (I+E; i.e. *in vitro *with endotoxin) or without (I; i.e. *in vitro*) stimulation *in vitro *(stimulation obtained in culture in the presence of 1 μg/ml endotoxin). CTRL, control group; LPS, animals that received LPS alone; LPS+aPC, animals that received LPS and aPC; aPC, animals that received aPC alone. *n* represents the number of rabbits. *p < 0.05 versus CTRL group; ^§^*p *< 0.05 versus I.

aPC alone did not modify monocyte TF expression at D5 (Figure 1). LPS administration increased monocyte TF expression in both unstimulated (I; *in vitro*) and stimulated (I+E; *in vitro *with endotoxin) cells when compared to monocytes taken in CTRL animals. In unstimulated monocytes, treatment with aPC in septic animals failed to blunt TF expression. In stimulated cells, the same level of TF expression was observed in the LPS+aPC group when compared to the LPS group, suggesting the ability to respond to further endotoxin stimulation.

### *In vitro *vascular reactivity

#### Vascular contraction

The maximal vasoconstrictor response to PE was not significantly different between groups (data not shown).

**Figure 2:**
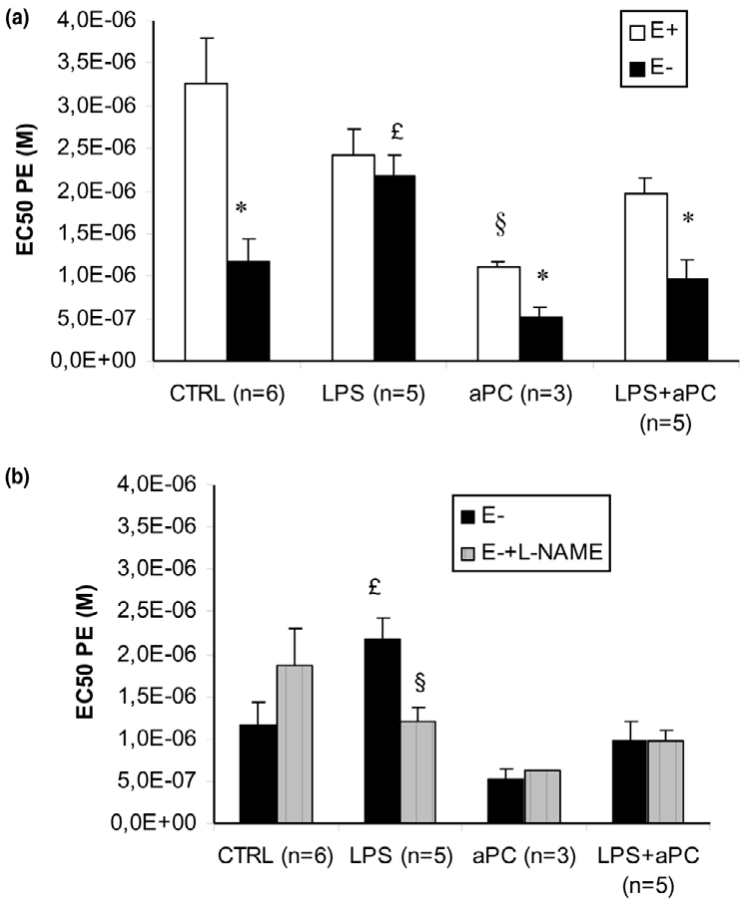
Phenylephrine (PE) concentration eliciting 50% of maximal constriction response (EC50) in different groups. CTRL, control group; LPS, animals that received LPS alone; LPS+aPC, animals that received LPS and aPC; aPC, animals that received aPC alone. *n* represents the number of rabbits. **(a) **Aortic rings in the presence of endothelium (E+) and in the absence of endothelium (E-). *p < 0.05 versus E+; ^§^*p *< 0.05 versus CTRL E+; ^163^*p *< 0.05 versus CTRL E-. **(b) **Aortic rings (E-) incubated with or without N^G^-nitro-L-arginine methyl ester (L-NAME). ^§^*p *< 0.05 versus LPS E-; ^163^*p *< 0.05 versus CTRL E-.

LPS significantly modified sensitivity to PE at D5. Indeed, PE EC_50 _of rings with endothelium (E+) and rings without endothelium (E-) were similar, suggesting endothelial dysfunction, when they were significantly different in the CTRL group (Figure 2a). aPC treatment in LPS animals restored the difference in sensitivity between E+ and E- aortic rings (LPS+aPC group). In the aPC group, there was persistence of endothelium-dependent contraction modulation: PE EC_50 _was different in E+ and E- aortic rings, similar to the CTRL group (Figure 2a). PE EC_50 _was significantly lower in E+ rings from the aPC group compared to E+ from the CTRL group.

In E- rings, LPS decreased the sensitivity to PE (versus the CTRL group). This difference was abolished after *in vitro *incubation with L-NAME, and was not observed in E- rings from aPC-treated rabbits (not significant, LPS+aPC group versus LPS group) (Figure 2b). No difference in sensitivity to PE of E- rings was observed between the aPC group compared to the CTRL group (Figure 2b).

#### Endothelium-dependent and endothelium-independent relaxation

Maximal endothelium-dependent receptor-dependent relaxation in response to ACh (Emax) was 78.3 ± 0.4% in the CTRL group. This response was altered by LPS administration (Emax = 50.0 ± 6.1%, *p *< 0.05 versus CTRL group). aPC treatment failed to restore ACh-induced vascular relaxation in septic rabbits (Emax = 33.5 ± 4.0%; *p *< 0.05 versus CTRL group).

Endothelium-dependent receptor-independent relaxation in response to calcium ionophore A23187 was not modified between groups (data not shown). A similar observation was recorded for endothelium-independent relaxation in response to sodium nitroprusside (data not shown).

#### Immunohistochemical staining of vascular endothelium

**Figure 3:**
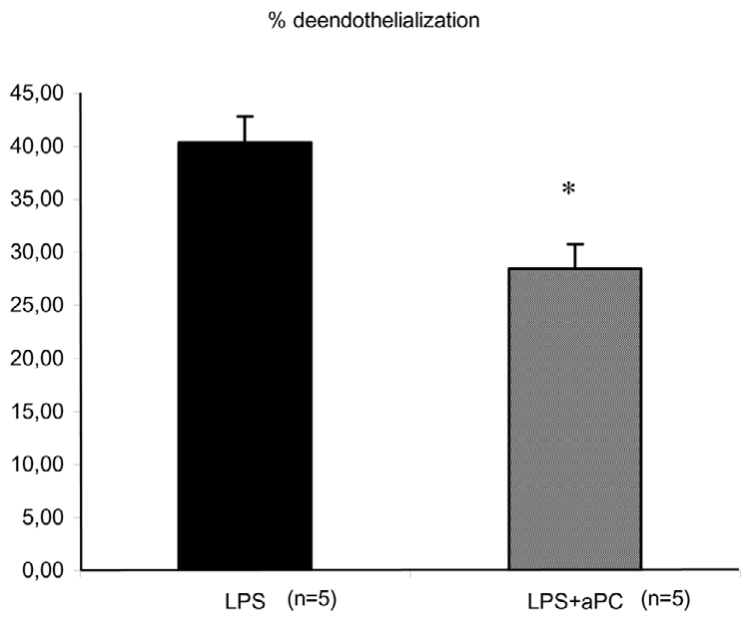
Quantification of abdominal aorta endothelial injury surface area by immunohistochemical study in endotoxic rabbits. LPS, animals that received LPS alone; LPS+aPC, animals that received LPS and aPC. n represents the number of rabbits. *p < 0.05 LPS+aPC group versus the LPS group.

For the sham groups (CTRL and aPC groups), endothelial cells stained by immunohistochemical label (PECAM1/CD31) appeared intact (Figure 3). LPS induced three types of endothelial cell injury: subendothelial vacuolization, detachment of endothelial cells and endothelial denudation. In the LPS group, the percentage of injured endothelium accounted for 40.4 ± 2.4% of total endothelial surface area in the abdominal aorta at D5 (*p *< 0.05 versus CTRL group). aPC treatment in LPS animals reduced these lesions, resulting in a surface area of endothelial injury of 28.5 ± 2.3% (*p *< 0.05 versus LPS group).

## Discussion

In the present study, we report that aPC is able to prevent endothelial morphological injuries and to increase the sensitivity to the vasoconstrictor agent PE in a well-documented rabbit endotoxin-induced shock model [7-12]. This was not associated with any effect on monocyte TF expression, endothelium-dependent relaxation in response to ACh, or mortality.

Our mortality rate was similar in LPS and LPS+aPC animals (16.7%). Because our model is a low mortality rate model, explanations about the absence of the effect of aPC on mortality cannot be drawn from this study. Taylor and colleagues [22] found a decreased mortality rate in septic baboons treated with aPC and Roback and colleagues [23] showed an increased survival rate in rabbits with LPS-induced meningitis that were treated with aPC. A main difference between our study and these two previous studies is that in the latter aPC was administered before LPS challenge whereas we decided to give aPC 1 hour after LPS injection. Another explanation may be related to the method of administration of aPC in our model; we injected aPC as a single bolus but it was administered as a continuous infusion in the two previous studies [22,23]. It has been shown that the half-life of aPC is decreased when administered to species different from human [17]. This suggests that its action might not be sustained over time, resulting in an absence of efficacy when LPS-induced effects have progressed. This is consistent with the fact that we did not observe improvement of either the *in vivo *or the arterial blood gas parameters of endotoxinic animals treated with aPC. We did not, however, assess the plasma level of aPC in our study. This assessment is not easily obtained in rabbit. During laparotomy for abdominal aorta extraction, we did not observe any organ haematomas or haemorrhage. We did not see any difference in either haematocrit or haemoglobin levels between endotoxinic animals treated or not with aPC. This suggests, as reported in the literature [24], that aPC does not cause bleeding or haemodilution.

A recent study reports that aPC binding to EPCR is a prerequisite to its action on mortality [25]. When aPC binds to EPCR, it activates a signaling pathway leading to inhibition of NF-κB expression and pro-inflammatory cytokines via the induction of protease activated receptor-1. Inadequate binding of aPC to EPCR could explain the absence of the anti-inflammatory effect and be responsible for the persistence of monocyte TF expression. This could be due to differences between the species used. Not administering aPC as an infusion and its short half-life could also explain the persistence of the inflammatory syndrome associated with persistence of monocyte TF expression at D5 after LPS bolus injection. Besides these pharmacokinetic considerations, our results are corroborated by a recent pharmacodynamic *in vivo *study using an acute human endotoxemia model [26]. This model allows studying the *in vivo *pharmacodynamics of drugs with anticoagulant or anti-inflammatory properties [27-29]. The authors concluded that aPC failed to decrease LPS-induced monocyte TF expression and failed to have any anti-inflammatory effects. They emphasized that the model used was an inadequate severe sepsis model with concentrations of aPC that remained above the pathological threshold. In the same way, studies that demonstrated an effect on monocyte TF expression were performed using *in vitro *experiments with supraphysiological concentrations of aPC [30-32]. Under these conditions, aPC might have acted as an anti-apoptotic agent [1,33]. This anti-apoptotic effect has been demonstrated in a human model of ischemic brain [34]. The anti-apoptotic signaling pathway of aPC might be different from that for NF-κB expression modulation.

A previous study demonstrated that aPC also has an anti-apoptotic effect on human endothelial cell cultures exposed to LPS [32]. By using immunohistochemical staining, we demonstrated that aPC restores and avoids prolonged vascular endothelial cell injury induced by endotoxinic shock. This result is in agreement with recent results [35], but the mechanism remains unclear. This was associated with improvement of endothelial cell function; in particular, aPC restores endothelium-dependent sensitivity to PE. This is in accordance with contractile induction of endothelial modulation. Indeed, as previously reported [7-12], LPS was responsible for the loss of the endothelium-dependence of PE sensitivity of aortic rings (the EC_50 _PE was similar between E+ and E- aortic rings in the LPS group). The sensitivity of smooth muscle cells to PE was decreased in aortic rings after LPS injection (the EC_50 _PE of E- rings from the LPS group was higher than the EC_50 _PE of E- rings from the CTRL group). This was restored by *in vitro *incubation with L-NAME, an inhibitor of NOS, suggesting the presence of iNOS in smooth muscle cells. In the present study, aPC treatment restores the endothelium-dependent sensitivity to PE in LPS-treated aortic rings (as observed in the CTRL group). This may result from reduced iNOS expression in smooth muscle cells. It has been recently demonstrated that aPC could inhibit excessive production of NO [35]. Moreover, we previously reported that monocyte TF expression may inhibit endothelial function [10,36]. Our results on restoration of PE sensitivity by aPC in spite of persistence of monocyte TF expression are in agreement with a recent study demonstrating that aPC has vascular protective effects independent of its action on coagulation [37]. Our results for the contractile response to PE, especially the increased sensitivity of aortic rings to PE when aPC is administered to non-endotoxinic animals (the EC_50 _of E+ rings of the aPC group is lower than that of E+ rings of the CTRL group, demonstrating an increased sensitivity to PE; Figure 2a), are consistent with a recent publication demonstrating that aPC improved vascular tone in septic patients [38].

Despite protective effects on endothelial structure and PE sensitivity, aPC failed to restore endothelium-dependent relaxation in response to ACh. These results suggest that the protective effect on endothelial function pertains to the PE signaling pathway. In our study, endothelium-altered relaxation specifically involves ACh, but not the endothelium-dependent receptor-independent agent calcium ionophore A23187. This suggests that an alteration in ACh receptor-NOS coupling and/or a reduced production of endothelium-derived NO causes this attenuated endothelium-mediated vasorelaxation. Another explanation may be the absence of any effect of aPC on similar pathways leading to expression of NF-κB and TF. This could result, at least in part, in a modified endothelial aPC signaling pathway due to EPCR dysfunction [15] or abnormal binding of aPC to EPCR. A recent study confirms our result on ACh-induced relaxation. The authors demonstrated that aPC did not relax norepinephrine-increased vascular tone in rabbit thoracic aorta [39].

## Conclusion

We demonstrate that aPC increases the sensitivity of aortic rings to the vasoconstrictor agent PE and restores endothelial modulation in the PE response. This was associated with decreased endothelial injury in endotoxin-treated animals. These results suggest that aPC may preserve endothelial structure via an anti-apoptotic effect. It failed to restore ACh-induced relaxation, suggesting that aPC probably acts differently in the relaxant and contractile signaling pathways. aPC did not modify monocyte TF expression. This suggests that aPC may act differently on monocyte TF expression or ACh receptor-NOS coupling. This could be caused by the lack of binding of aPC to EPCR, explaining its lack of effect on the NF-κB pathway, the inflammatory process, monocyte TF expression, and mortality.

## Key messages

• 	In our model of endotoxinic shock, aPC increases the sensitivity to vasoconstriction.

• 	aPC restores endothelial modulation in the PE response.

• 	aPC has protective effects on endothelial structure, probably via an anti-apoptotic effect.

• 	aPC did not modify coagulation activation, defined in our model as monocyte tissue factor expression.

• 	aPC failed to restore ACh-induced relaxation, suggesting that it probably acts differently in the relaxant and contractile signaling pathways.

## Abbreviations

ACh = acetylcholine; aPC = activated protein C; CTRL = control; E- = without endothelium; E+ = with endothelium; EC_50 _= concentration of agonist causing half-maximal contraction or relaxation; EPCR = endothelial protein C receptor; iNOS = inducible nitric oxide synthase; L-NAME = N^G^-nitro-L-arginine methyl ester; LPS = lipopolysaccharide; NF = nuclear factor; NO = nitric oxide; PBS = phosphate buffer saline; PC = protein C; PE = phenylephrine; TF = tissue factor.

## Competing interests

The authors declare that they have no competing interests (aPC was provided by Eli-Lilly).

## Authors' contributions

All the authors contributed to the elaboration of the protocol, its feasibility and the preparation of the manuscript. EW and MEC performed the vasoreactivity study, blood gas analysis and immunohistochemical staining. EW and GL were responsible for the statistical analysis. DC and BJ performed the isolation of monocytes, the determination of TF expression and the study of hematological and coagulation variables. RB, BT and BV participated in the elaboration of the protocol, and the preparation and correction of the manuscript.

## Figures and Tables

**Table 1:** Hematological variables at day 5

Group	Leukocytes (10^3^/mm^3^)	Neutrophils (10^3^/mm^3^)	Lymphocytes (10^3^/mm^3^)	Monocytes (10^3^/mm^3^)	Hemoglobin (g/l)	Hematocrit (%)	Platelets (10^3^/mm^3^)
CTRL (*n* = 6)	7.1 ± 1.0	3.65 ± 1.09	2.64 ± 0.42	0.37 ± 0.08	12.5 ± 0.6	38.6 ± 1.1	365 ± 54
aPC (*n* = 4)	4.2 ± 0.6	1.98 ± 0.30	1.70 ± 0.32	0.20 ± 0.06	13.0 ± 0.4	39.8 ± 0.8	254 ± 10
LPS (*n* = 5)	6.3 ± 0.3	2.58 ± 0.31	3.09 ± 0.23	0.49 ± 0.08	11.3 ± 0.2	36.7 ± 1.0	495 ± 120
LPS+aPC (*n* = 5)	7.6 ± 1.0	2.96 ± 0.57	3.62 ± 0.76	0.65 ± 0.09	11.3 ± 0.3	35.5 ± 1.0	478 ± 65

Groups: aPC, animals that received aPC alone CTRL, control; LPS, animals that received LPS alone; LPS+aPC, animals that received LPS and aPC. *n* represents the number of rabbits.

**Table 2:** Coagulation variables at day 5

Group	PI (%)	Fibrinogen (g/l)	Factor II (%)	Factor V (%)	Factor VII+X (%)
CTRL (*n* = 6)	88 ± 7	3.8 ± 0.8	101 ± 8	80 ± 6	111 ± 10
aPC (*n* = 4)	81 ± 3	3.2 ± 0.2	90 ± 3	70 ± 2	100 ± 8
LPS (*n* = 5)	105 ± 2	9.8 ± 1.4^a^	162 ± 14^a^	138 ± 18	210 ± 22^a^
LPS+aPC (*n* = 5)	102 ± 5	8.6 ± 1.8	154 ± 21	123 ± 24	173 ± 27

Groups: aPC, animals that received aPC alone; CTRL, control; LPS, animals that received LPS alone; LPS+aPC, animals that received LPS and aPC. *n* represents the number of rabbits. ^a^*p *< 0.05 versus CTRL. PI, prothrombin index.
